# Our Bureau of Information

**Published:** 1912-02-10

**Authors:** 


### OUR BUREAU OF INFORMATION.

### The Limits of the Bureau.

Our correspondence has already shown us that some of our readers, while realising the general intention of the Bureau, do not grasp its limitations. They do not understand the things which we can *not* do. Thus, although we have specifically stated that we cannot recommend special practitioners, letters come asking us to name a doctor skilled in this or that disease. If our readers are at a loss, they can consult their local medical attendant; and if he cannot advise them, they can consult "Medical Homes for Private Patients" or Burdett's "Hospitals and Charities," which latter book they are almost certain to find in their public library. Let them look up the hospitals dealing with the particular kind of disease which they want treated, and choose one of the honorary staff of one of these for a consultant. They can scarcely go wrong if they act on this principle.

We have also had letters dealing with all manner of human derelicts. We welcome such letters, though it is only here and there that one of them may, from a previous career, enable us to indicate some possible form of help; but such cases are rare, and it must be remembered that our particular mission is to help the sick, and that we cannot undertake the functions of a general charity organisation. Neither can we—as some correspondents desire—get one of our staff to examine a patient who thinks his case has not been properly managed by his own doctor and advise as to a different course of treatment. It is not easy for a doctor who does not see a patient until his limb has been taken off to decide that amputation was unnecessary. The *casus belli* is no longer there! Put in bald print the idea is absurd, yet our advice has been asked in such a case.

It has been made clear that we do not advertise nursing-homes unless they are prepared to give us sufficient guarantee as to their efficiency, and also to pay our registration fee. The first rule alone has been sufficient to keep out of our list far more institutions than have been included. But it must be understood that the regulation affects not only nursing-homes but all correspondents whose object is business—not necessarily pecuniary profit. For those who want posts in the institutional world the course is plain. They can apply direct to suitable places, or can advertise their wishes. Therefore for all correspondents who wish to obtain posts we make the same rule as for nursing-homes. They must pay a registration fee of five shillings. For this fee we will advertise their wants in two consecutive issues, and will also keep their names and what they want on a list, to which we will refer when anyone wants such services. We trust that our readers will see the reasonableness of this rule.

### NEEDS AND HELPERS.

### HOME FOR A CHILD WITH DISEASE OF THE EAR.

"E. L. C." writes: Can you help me to find a home for a boy, five-and-a-half years, who is suffering from disease of the middle ear, of about three years' standing, accompanied by discharge? The mother is a deserted wife, and is in domestic service. She has been paying 3s. 6d. a week to a respectable woman to take care of the child, and the latter has done her best, but cannot provide what is needed—good food, good air, and daily treatment under medical supervision. If these could be provided for a year or two the case might, the doctor thinks, be cured. Is there any home—by preference under a trained nurse—where the boy could be taken at this rate of payment?

(The matron of the "Ida" Convalescent Home for Children, Filey Road, Scarborough, answers: This Home takes cases requiring some months' treatment if there is hope of a cure. The fees are 2s. 6d. a week during the winter months, and 3s. 6d. in the summer, with a subscriber's ticket for each month. The matron is a London hospital certificated nurse, and there are three doctors on the staff.)

"Southend" writes: My wife, who is 52 years of age, has been suffering more or less for eighteen years from what she is now told is tubercular disease of the hip. She is in perfect health otherwise, but the limb will not bear any exertion without causing great pain. Is there any institution where she could be treated?

"F. W. G., Gosport," writes: Can you furnish me with any information of where I should be likely to obtain a situation in a laboratory of a hospital, where I could further increase my knowledge of laboratory work? I may mention that salary would be optional.

("Hospital Official" answers: I would suggest that your inquirer should send particulars of his experience to various hospitals, and ask for any vacancy that may arise. I should particularly advise him to apply to the Middlesex, St. Mary's, and the Cancer Research Fund. I may say that the pay is low, but assistants are supposed to be improving their knowledge with a view to obtaining higher posts.)

"Borage" writes: I am ill with that unexplainable misery of neurasthenia. I cannot work and am without means, so am an extra burden in a home that I helped to keep going. The doctor says I should be better away, and that I must eat plenty of good food to get well. But while I can earn nothing plenty of food and change of air are not possible. Are there no free hospitals or Homes where I might have a chance of building up my body and nervous system, so as to enable me to take up my work again? I would give no trouble, and could do light duties in return for a change of scene and good food. I am a governess by profession, and am 25 years of age.

"Orman" writes: Can any of your correspondents tell me of a Home where a patient can be received for about one guinea a week?

### Our List of Approved Homes.

We are pleased to include in our list of approved Homes the following:—

"Woodmancote," Woodland Vale Road, St. Leonards-on-Sea. (Tel. 611 Hastings.) Matron, Miss Reinberg. All ordinary cases, chronics, and convalescents. No infectious or phthisis cases received. Good operating theatre. Terms from 3 guineas a week.

13 St. Vincent's Road, Westcliff-on-Sea. Principal, Mrs. Steele. Senile and mild mental cases.

### Rules for Correspondents.

Everyone who uses or wishes to help our Bureau of Information must be kind enough to observe the following rules:—

(1) Postal replies cannot be given. Exception will only be made in very special cases at the Editor's discretion.

(2) Queries or answers relating to different cases must be written, or preferably typed, on separate sheets of paper, and the writing must never be crossed.

(3) Questions and replies received not later than Wednesday will, as far as possible, be dealt with in our issue of the following Saturday week.

(4) Letters must have attached the name and address of the sender, with a pseudonym for publication if preferred.

(5) Applications referring to non-dependents where the need relates to business or employment must be accompanied by a postal order for 5s., when the requirement shall have publicity in these columns.

(6) All applicants for insertion in our list of Approved Homes must send a registration fee of five shillings. The omission of this leads to delay and gives avoidable trouble.

(7) *All communications* for this department *must be accompanied by the coupon* to be found at the bottom of the third page of the cover on the inside, and should be addressed to the Editor of the Bureau of Information, c/o The Hospital, 29 Southampton Street, Strand, London, W.C.

